# Mutation analysis at codon 838 of the *Guanylate Cyclase 2D* gene in Spanish families with autosomal dominant cone, cone-rod, and macular dystrophies

**Published:** 2011-04-29

**Authors:** Maria Garcia-Hoyos, Carmen Laura Auz-Alexandre, Berta Almoguera, Diego Cantalapiedra, Rosa Riveiro-Alvarez, Miguel Angel Lopez-Martinez, Ascension Gimenez, Fiona Blanco-Kelly, Almudena Avila-Fernandez, Maria Jose Trujillo-Tiebas, Blanca Garcia-Sandoval, Carmen Ramos, Carmen Ayuso

**Affiliations:** 1 Genetics Department, Instituto de Investigacion Sanitaria-Fundacion Jimenez Diaz (IIS-FJD), Madrid, Spain; 2 Centro de Investigacion Biomedica en Red de Enfermedades Raras (CIBERER), ISCIII, Madrid, Spain; 3 Ophthalmology, Instituto de Investigacion Sanitaria-Fundacion Jimenez Diaz (IIS-FJD), Madrid, Spain

## Abstract

**Purpose:**

Heterozygous mutations around codon 838 of the guanylate cyclase 2D (*GUCY2D*) gene have recently been associated with more than a third of autosomal dominant macular dystrophy patients. The aim of our study was to evaluate the prevalence of these mutations in Spanish families with autosomal dominant cone, cone-rod, and macular dystrophies.

**Methods:**

Mutation analysis was performed by PCR amplification of exon 13 of *GUCY2D* and subsequent restriction analysis. To confirm the results, automatic sequencing analysis was also performed.

**Results:**

Among the 22 unrelated Spanish families included in the study, we found two associated disease mutations at codon 838 of the *GUCY2D* gene, one of which had not been previously described (p.R838P). This novel mutation exhibited phenotypic variability.

**Conclusions:**

The prevalence of mutations around codon 838 of *GUCY2D* in our group of families (9.09%) is lower than that previously reported in other populations. However, the discovery of a novel mutation at codon 838 further suggests that this locus is a mutation hotspot within the *GUCY2D* gene, and confirms the importance of analyzing this codon to characterize molecularly these autosomal dominant retinal disorders.

## Introduction

Cone-rod dystrophy (CORD) and cone dystrophy (COD) are a subgroup of inherited retinal dystrophies characterized by progressive loss of photoreceptor function. In CORD, there is a progressive loss of cone photoreceptor function followed by gradual loss of rod photoreceptor function and retinal degeneration. In COD however, cone function decreases progressively from its onset, though rod function is well preserved until the late stages of the disease. The predominant symptoms of these disorders are decreased visual acuity, bright light aversion, and decreased sensitivity in the central visual field, sometimes followed by progressive loss of peripheral vision and night blindness [1-6].

CORD and COD are genetically heterogeneous, with described dominant, recessive and X-linked inheritance patterns. To date, ten loci for autosomal dominant CORD and COD have been identified: cone-rod dystrophy 4 (*CORD4*) [7], retinal cone dystrophy 1 (*RCD1*) [8], arylhydrocarbon-interacting receptor protein-like 1 (*AIPL1*) [9], cone-rod homeobox-containing gene (*CRX*) [10], guanylate cyclase activator 1A (*GUCA1A*) [11], guanylate cyclase 2D (*GUCY2D*) [12], phosphatidylinositol transfer protein membrane-associated 3 (*PITPNM*) [13], protein regulating synaptic membrane exocytosis 1 (*RIM1*) [14], semaphorin 4A (*SEMA4A*) [15] and Homolog of *C. elegans* 119 (*UNC119*) [16].

The previously reported prevalence of the *GUCY2D* gene mutations around codon 838 in CORD and COD is about 35%. Heterozygous missense mutations at codons 837, 838, or 839 in the *GUCY2D* gene, which produce a gain of function, have been shown to cause autosomal dominant CORD and COD [12,17-21], whereas homozygous or compound heterozygous mutations, which produce loss of function, cause Leber congenital amaurosis (LCA) [22-24].

The *GUCY2D* gene is located in 17p13.1 (LCA1/CORD6) [18,25]. It is 16 kb long and encodes a protein 1,103 amino acids long. The 20 exons identified in this gene code for a photoreceptor-specific protein, retinal guanylyl cyclase-1 (RetGC-1), mostly located in the marginal region of the cone’s outer segments. RetGC proteins play an important role in restoring photoreceptor sensitivity due to their involvement in the synthesis of cyclic guanosine monophosphate (cGMP) from guanosine triphosphate (GTP). Light stimulates the degradation of cGMP, causing the closing of cation channels, which results in a reduction of the Na^+^ and Ca^2+^ concentration, cell hyperpolarization, and slowing of neurotransmitter release. At a lower concentration, the Ca^2+^ binds guanylate cyclase activator proteins (GCAPs) and stimulates the RetGCs, and in consequence, the cGMP level is restored. As a result, the cation channels reopen and photosensitivity is restored to the cell [26].

**Figure 1:**
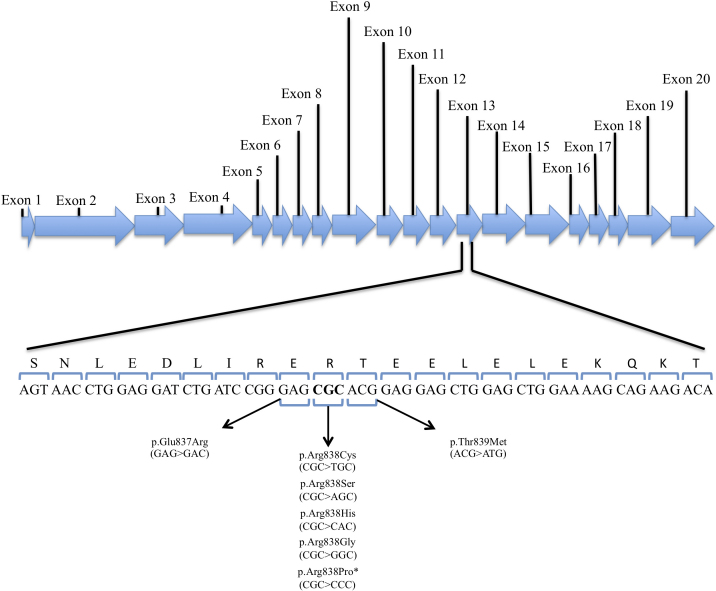
Reported mutations in a fragment of the primary structure of the retinal guanylyl cyclase-1 (RetGC-1) protein, encoded by exon 13 of Guanylate Cyclase 2D (*GUCY2D*). This figure shows the different mutations that have been previously described in codons 837, 838, and 839 of the *GUCY2D* gene. The asterisk indicates the novel p.Arg838Pro mutation described in this manuscript. The shadowed area corresponds to the Hemophilus hemolyticus (HhaI) recognition site.

To our knowledge, all *GUCY2D* mutations identified so far in CORD or COD patients are located at codon 838 or the two adjacent ones (Figure 1) [12,17-21]. The aim of this study was to analyze the prevalence of *GUCY2D* mutations clustered at codon 838 in Spanish patients with CORD, COD, and autosomal dominant macular dystrophy (adMD).

## Methods

### Selection of patients

A total of 22 unrelated Spanish patients clinically diagnosed with CORD-COD or adMD and with a family history consistent with an autosomal dominant mode of inheritance were included in this study.

After ophthalmologic examination, adMD patients were classified according to the following criteria: fundus compatible with MD (atrophy and/or hyperpigmentation spots restricted to the macular region, drusen-like fundus, yellow spots), an electroretinogram (ERG) showing reduction of cone signals but normal rod signals, progressive loss of central vision (central scotoma), progressive reduction of visual acuity, and dyschromatopsia.

Applying these criteria and based on clinical examination (ERG and fundus photographs), 10 patients were diagnosed with CORD or COD, whereas 12 patients were classified as having adMD. The first group (CORD or COD) included cases with decreased central vision, abnormal color vision, abnormal ERG, and fundus pigment changes. Ganzfeld electroretinography was recorded according to International Society for Clinical Electrophysiology of Vision (ISCEV) standards, using the UTAS 2000 system (LKC Technology, Gaithersburg, MD) and jet electrodes.

In the family with the novel mutation, seven affected members and four unaffected members were used for segregation analysis and clinical examinations.

In addition, 190 randomly selected DNA samples (380 chromosomes) from a healthy control population were analyzed to assess the frequency of sequence changes in the normal population.

Informed consent was obtained from all persons involved in the study, in accordance with the tenets of the Declaration of Helsinki (Seoul, 2008).

### Screening for mutations

DNA was extracted from peripheral blood leukocytes collected in EDTA tubes, in an automated DNA extractor according to manufacturer instructions (BioRobot EZ1; Qiagen, Hilden, Germany).

Exon 13 of the human *GUCY2D* gene, which includes codon 838 and surrounding codons, was directly amplified from genomic DNA using primers previously described [27] Forward primer: 5′CAG CTT TAC CAG CTT CCT TC 3′, melting temperature=56.9 °C; Reverse primer: 5′ GCA GGC AGT GAG GTC ACC TG 3′, melting temperature=64.4 °C). A sample of genomic DNA (100 ng) from patients or control individuals were used in a 25 µl reaction mixture containing 0.6 µM of forward and reverse primers, 24 µM of each dNTP, 1× PCR buffer, and 1 U of FastStart Taq DNA polymerase (Roche, Indianapolis, IN). After an initial denaturation of 95 °C for 5 min, 30 cycles were performed at 95 °C for 30 s, 63 °C for 20 s, and 74 °C for 50 s, with a final extension step of 74 °C for 5 min.

The PCR products (278 base pairs length) were digested with HhaI according to the manufacturer instructions (New England BioLabs, Beverly, MA) and resolved by electrophoresis in 5% metaphor agarose (Lonza, Rockland, ME). Wild-type samples produce two fragments of 130 bp and 150 bp, but the restriction target site (5′-… GCGC …-3′) in exon 13 of *GUCY2D*, which lies between the last nucleotide of codon 837 and the last nucleotides of codon 838 (both included), is destroyed by the previously reported mutations at these two codons (Figure 1).

To confirm the results obtained (positive and negative for restriction enzyme digest), PCR products were also sequenced using the BigDye Terminator v. 1.1 Cycle Sequencing kit (Applied Biosystems, Foster City, CA) in the ABI 3130*xl* Genetic Analyzer (Applied Biosystems) and analyzed with the Sequencing Analysis v. 5.2 software package (Applied Biosystems).

### Haplotype analysis

**Figure 2:**
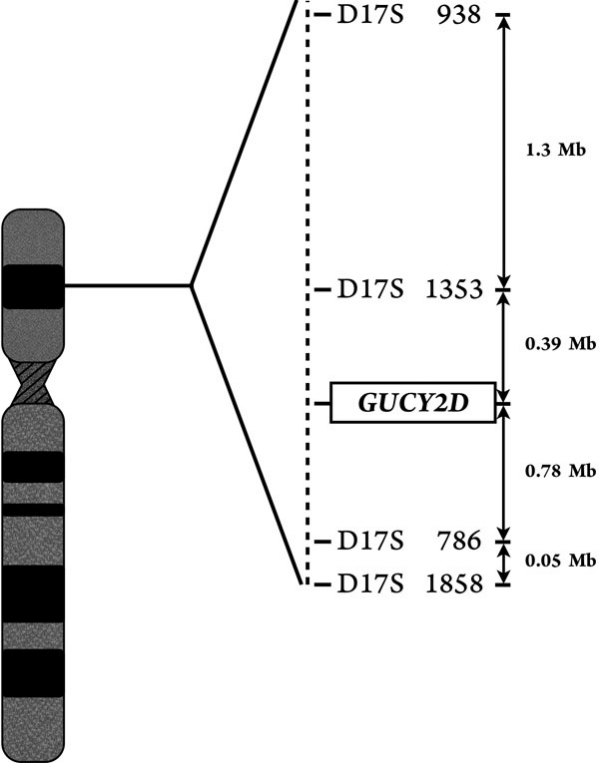
Figure shows a map of the CORD6 region. Distance between markers and the locus CORD6 are indicated in bp.

To assess the role of the *CORD6* locus, haplotype analysis was performed using four polymorphic markers with a strong link to this locus: D17S938, D17S1353, D17S786, and D17S1858 (Figure 2). For all four markers, each forward PCR primer was fluorescently labeled and separately amplified by PCR in a total volume of 15 µl containing 100 ng of genomic DNA, 125 µM of each dNTP, 10 pmol of each primer (forward and reverse), 1× Taq DNA polymerase buffer (500 mM Tris/HCl, 100 mM KCl, 50 mM [NH_4_]_2_SO_4_, 20 mM MgCl_2_), and 0.6 U of FastStart Taq DNA Polymerase (Roche). After denaturation at 95 °C for 5 min, PCR was performed in a GeneAmp PCR System 2700 (Applied Biosystems) for 10 cycles at 94 °C for 30 s, 55 °C for 30 s, and 72 °C for 90 s, and then at 15 cycles at 89 °C for 30 s, 55 °C for 30 s, and 72 °C for 90 s, with a final extension time of 30 min at 72 °C. For the genotyping process, PCR products were electrophoresed in an ABI 3130*xl* Genetic Analyzer (Applied Biosystems) and analyzed with the GeneMapper v. 4.0 software package (Applied Biosystems).

## Results

We identified the mutation associated with the disease in two of the 22 (9.09%) unrelated Spanish families that were included in this study because of their suspected clinical diagnosis. In all cases, the additional sequencing analysis of exon 13 of the human *GUCY2D* gene confirmed the results obtained using restriction analysis.

**Figure 3:**
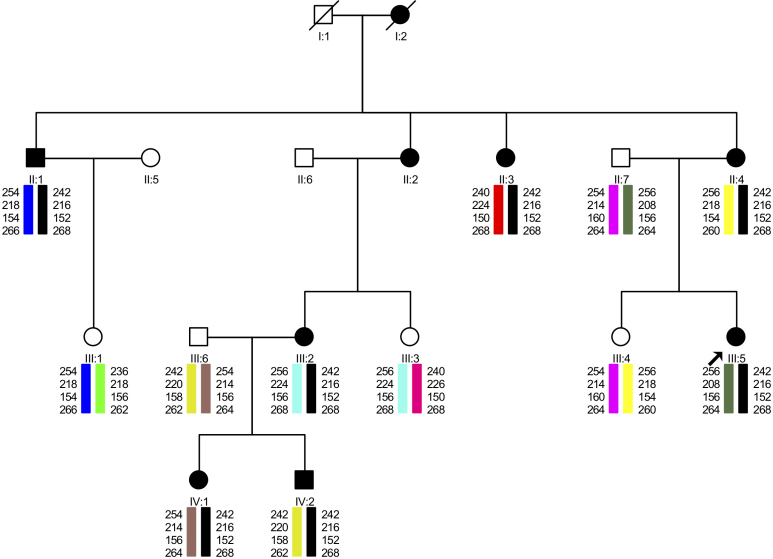
Results of the haplotype analysis performed in a family with the p.R838P mutation in Guanylate Cyclase 2D (*GUCY2D*). The figure also shows the status of genotyped individuals for mutation p.R838P, where “+” is used for normal alleles and “–” is used for mutated alleles.

**Figure 4:**
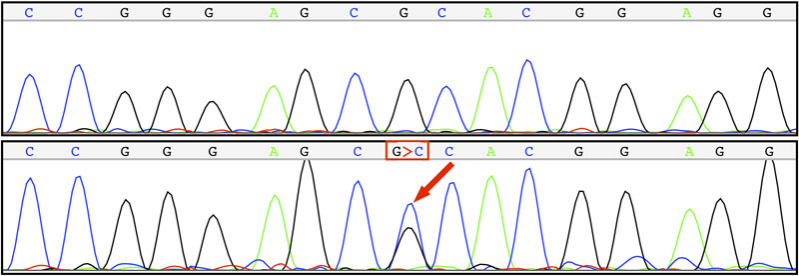
A heterozygous mutation (Guanine-to-Citosine; G>C) results in an arginine-to-proline mutation at codon 838 of the Guanylate Cyclase 2D (*GUCY2D*) gene.

**Figure 5:**
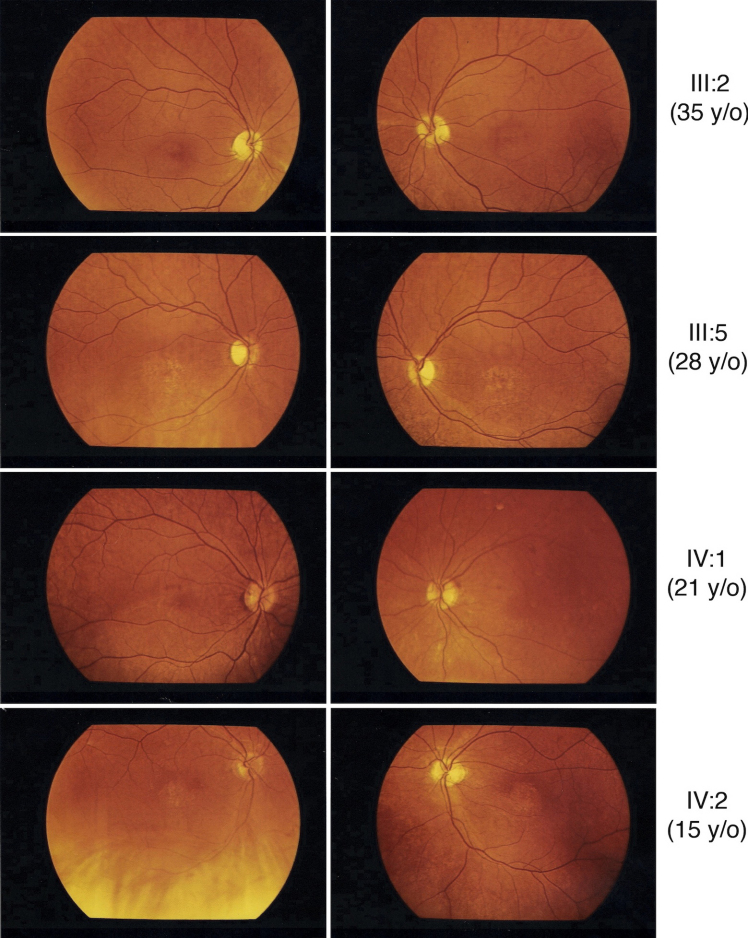
Fundus photograph of five different patients of the families with p.R838P mutation in *GUCY2D.* Older patients had a more severe phenotype compared to the younger generation, and to the Goldmann kinetic perimetric fields of patients III:5 and IV:2. Fundus description for these five patients has been summarized in Table 1.

One proband carried a novel c.2513G>C (p.R838P) mutation that segregated with the disease in this family, as all affected members presented this change which was absent in his family (Figure 3) and was absent in 380 control chromosomes (Figure 4). In the family with the novel mutation (p.R838P), the clinical phenotype associated with the disease was characterized both by its onset in the first decade of life and by the detection of a central scotoma. Nystagmus and reduced visual acuity were noticed in all affected family members, although no color vision abnormality or increased glare sensitivity were reported (Table 1). Within this family, older patients typically had a more severe phenotype than did the younger patients (Figure 5).

**Table 1:** Clinical features of the families with the p.R838P mutation in *GUCY2D.*

**Individual**	**Age**	**Visual acuity (RE/ LE)**	**Fundus findings**	**Central scotoma**	**Color vision**	**Nystagmus**
II:1	70	0.01/0.01	Macular degeneration	+++	Normal	+
II: 3	61	0.3/0.2	Macular degeneration	N/A	Normal	+
II:4	59	0.01/0.01	Macular degeneration Salt-and-pepper fundus appearance in the posterior pole	++	Normal	+
III:2	35	0.2/0.2	Tapetoretinal degeneration Salt-and-pepper fundus appearance	++	Normal	+
III:5	28	0.3/0.1	Discreet salt-and-pepper fundus appearance	++	Normal	+
IV:1	21	N/A	Salt-and-pepper fundus appearance	++	Normal	+
IV:2	15	0.1/0.1	Poorly distinguishable macula	+	Normal	+

In addition, another affected patient was heterozygous for the mutation c.2513G>A (p.R838H), previously reported by Weigell-Weber et al. [19]. This affected patient had gross central macular degeneration in both eyes, with bilateral central scotoma. The best-corrected visual acuity was 0.1 in each eye at the age of 59 years.

## Discussion

Since the aim of our study was to evaluate the prevalence of mutations at codon 838, we used a restriction enzyme digestion (RE) that could detect all possible mutations at codon 838 and also at codon 837, but not at the adjacent codon 839. Additional sequencing of exon 13 of GUCY2D confirmed that only the two mutations previously detected at codon 838 by using RE (c.2513G>C and c.2513G>A) were associated with the disease in our cohort of patients. For that reason, we proposed the use of RE as a quick first step to analyze our families.

Although a previous study reported mutations around codon 838 of *GUCY2D* associated with CORD or COD in more than a third of the patients [17], the prevalence of *GUCY2D* mutations around this codon was lower (9.09%) in our group of Spanish families.

Four different mutations have previously been reported as affecting codon 838: p.R838C [12], p.R838H [19], p.R838S [19], and p.R838G [17]. We report a new mutation at this particular locus, (p.R838P), which further indicates the importance of this codon and leads us to confirm that it is a mutational hotspot in the *GUCY2D* gene, associated with CORD, COD, and adMD disease.

At a lower concentration, the Ca^2+^ binding GCAPs stimulate the RetGC proteins. Activation of RetGC1 by GCAP1 involved dimerization of two RetGC1 monomers. The dimerization domain of RetGC-1 extends from amino acid 817 to 857, and this region is likely to adopt a coiled-coil structure [28]. Mutations at position 838 have been described as increasing the stability of the coiled-coil; hence, the mutant protein retains residual catalytic activity even at high calcium levels [27]. These mutations increase the affinity of RetGC-1 for GCAP1 and alter the Ca^2+^ sensitivity of the GCAP1 response, allowing the mutant to be stimulated by GCAP1 at higher Ca^2+^ concentrations than is the wild type [28]. This gain of function results in the maintenance of GMP levels, and consequently in a persistently high intracellular Ca^+2^ level. It is known that a persistent elevated calcium level in the cell tends to disrupt the membrane potential of the mitochondrial inner membrane, leading to the release of cytochrome C, with subsequent caspase activation and apoptosis [23]. This may be the mechanism, resulting from *GUCY2D* mutations, of photoreceptor degeneration in CORD or COD, and in adMD. While, heterozygous mutations in *GUCY2D* associated with CORD or COD have been described that cause a gain of function, individuals harbouring either homozygous or compound heterozygous GUCY2D loss-of-function mutations present a more severe disease, such as LCA is associated with being homozygous or compound heterozygous for *GUCY2D* loss-of-function mutations [12,22].

The phenotypes of the families with mutations at codons 837, 838, or 839 of *GUCY2D* appear to vary, depending on the specific mutation and the presence or absence of multiple mutations [17,27,29]. In our family with the novel mutation p.R838P, no color vision abnormalities or increased glare sensitivity were reported, though affected members showed reduced visual acuity and a central scotoma.

We believe that this report highlights the importance of codon 838 of GUCY2D as a mutation hotspot associated with CORD or COD and with adMD. In addition, genetic studies of families clinically diagnosed as having CORD, COD, or adMD are important for performing correct genetic counseling.

Moreover, knowledge of the molecular mechanism of these diseases would permit the development of new potential therapies, such as gene therapy. Mutations at codon 838 of *GUCY2D* are related to autosomal dominant disease; therefore, silencing the mutant allele using allele-specific interference RNA could be used in this case to partially rescue the protein function [30-32].
